# Severe below-maintenance feed intake increases methane yield from enteric fermentation in cattle

**DOI:** 10.1017/S0007114519003350

**Published:** 2020-03-25

**Authors:** J. P. Goopy, D. Korir, D. Pelster, A. I. M. Ali, S. E. Wassie, E. Schlecht, U. Dickhoefer, L. Merbold, K. Butterbach-Bahl

**Affiliations:** 1 Mazingira Centre, International Livestock Research Institute (ILRI), Mazingira, Nairobi 30709, Kenya; 2 University of Melbourne, Melbourne, Australia; 3 Science and Technology Branch, Agriculture and Agri-Food Canada, Québec, QC, Canada; 4 Animal Husbandry in the Tropics and Subtropics, University of Kassel/University of Goettingen, Witzenhausen 37213, Germany; 5 Animal Nutrition and Rangeland Management, Institute of Agricultural Sciences in the Tropics, University of Hohenheim, Stuttgart 70599, Germany; 6 Institute of Meteorology and Climate Research, Atmospheric Environmental Research, Karlsruhe Institute of Technology, Garmisch-Partenkirchen, Germany

**Keywords:** *Y_m_*, Sub-maintenance feeding, Sub-Saharan Africa, Enteric fermentation

## Abstract

The relationship between feed intake at production levels and enteric CH_4_ production in ruminants consuming forage-based diets is well described and considered to be strongly linear. Unlike temperate grazing systems, the intake of ruminants in rain-fed tropical systems is typically below maintenance requirements for part of the year (dry seasons). The relationship between CH_4_ production and feed intake in animals fed well below maintenance is unexplored, but changes in key digestive parameters in animals fed at low levels suggest that this relationship may be altered. We conducted a study using Boran yearling steers (*n* 12; live weight: 162·3 kg) in a 4 × 4 Latin square design to assess the effect of moderate to severe undernutrition on apparent digestibility, rumen turnover and enteric CH_4_ production of cattle consuming a tropical forage diet. We concluded that while production of CH_4_ decreased (1133·3–65·0 g CH_4_/d; *P* < 0·0001), over the range of feeding from about 1·0 to 0·4 maintenance energy requirement, both CH_4_ yield (29·0−31·2 g CH_4_/kg DM intake; *P* < 0·001) and CH_4_ conversion factor (*Y_m_* 9·1–10·1 MJ CH_4_/MJ gross energy intake; *P* < 0·01) increased as intake fell and postulate that this may be attributable to changes in nutrient partitioning. We suggest there is a case for revising emission factors of ruminants where there are seasonal nutritional deficits and both environmental and financial benefits for improved feeding of animals under nutritional stress.

The greatest determinants of enteric CH_4_ production in ruminants are the quantity and digestibility of feed ingested. The relationship between feed intake and daily CH_4_ production rate (MPR) has been determined experimentally over many years down to the present^(1–6)^. Whilst it has been found that there is variability in MPR between animals fed the same ration, and variability between days for the same animal^(7)^, it has also been demonstrated that there is a strong positive, linear relationship between MPR and the level of intake of a mainly forage diet that holds generally constant over a large range of intakes and types of forages^(8)^.

Sub-Saharan Africa has a ruminant livestock sector which is responsible for a disproportionally large level of anthropogenic greenhouse gas emissions in economies that are dominated by agriculture^(9)^, and there is a pressing need to comprehensively and accurately estimate these emissions. A key plank in the development of emission factors is the employment of accurate CH_4_ conversion factors (*Y*_*m*_: CH_4_ (MJ)/gross energy intake (MJ)). Equations developed by Charmley *et al.*^(8)^ have been derived from measurements of both *Bos taurus* and *Bos indicus* cattle consuming a wide range of tropical and temperate forages and thus may be considered more applicable to African cattle than equations derived solely from temperate regions. However, a significant limitation to the utility of these equations is that measurements have been conducted only on animals fed for production (i.e. at maintenance and above), whereas it has been recently demonstrated that cattle regularly experience episodes of significant seasonal weight loss in African smallholder systems^(10,11)^.

It has been established that higher CH_4_ yield (MY: g CH_4_/kg DM intake) is associated with longer mean rumen retention time (MRT) in sheep^(12,13)^, while both feed digestibility and MRT increase with declining intake in limit-fed cattle^(14)^. In contrast, the relationship between intake and MY in animals consuming feed at levels below maintenance requirements is not well understood and there is circumstantial evidence to suggest that the relationship may be altered when intake is very low. Several studies have demonstrated that MRT increases in ruminants fed food of a given quality well below maintenance, while apparent digestibility remains unchanged, or in some cases, actually declines^(15–18)^. Increasing the time digesta spends within the rumen may expose it to a kind of futile cycling, where material which may otherwise be enzymatically digested by the host is subjected to further microbial degradation in the rumen, resulting in higher production of volatile fatty acids, H^+^ and CH_4_, at the expense of microbial protein. Refermentation of digesta has been observed in sheep fed specialised diets^(19)^ and has been posited as a mechanism where sheep fed low-quality diets produced more CH_4_ than predicted^(20)^.

At present, the Intergovernmental Panel on Climate Change guidelines recommend adopting a *Y*_*m*_ of 6·3 % to estimate enteric emissions from cattle consuming a tropical forage-based diet, which has been substantially corroborated by the extensive study of Charmley *et al.*^(8)^. However, if below-maintenance intake results in a higher than expected MPR, this will have significant implications, both for inventory and for intervention options in systems where ruminants often can only access below maintenance rations for part of the year – as in Sub-Saharan Africa. Thus, this study investigated the relationship between feed intake, rumen kinetics, apparent digestibility and CH_4_ production in animals consuming diets at levels well below maintenance requirements. We hypothesised that MY and thus *Y*_*m*_ would increase with increasing severity of sub-maintenance feeding.

## Materials and methods

### Animals and experimental design

All animal procedures were carried out adhering to the international standards for animal care and use for scientific purposes, reviewed by the Institutional Animal Use and Care Committee of the International Livestock Research Institute permit no: IACUC-RC2016-11.

Boran (*B. indicus*) yearling steers (*n* 12; live weight (LW): 162·3 (sem 3·77) kg) were sourced from a commercial ranch in Lakipia County (Northern Kenya). Before the commencement of the trial, animals were treated with an anthelmintic, an acaricide wash, vaccinated for foot and mouth disease and clostridial diseases, ear tagged and placed under quarantine for 21 d as part of the standard induction procedure of the institution. Steers were housed in open individual pens (1·90 × 2·87 m), covered with shade-cloth sails, with clean water supplied *ad libitum* from automatic waterers.

The experimental design was a 4 × 4 Latin square with four levels of feeding (maintenance energy requirement (MER): 1·0 MER, 0·8 MER, 0·6 MER, 0·4 MER). Steers were stratified by LW and randomly allocated to the four treatment groups (three animals per group, with the individual animal being the experimental unit). The groups were maintained throughout the experimental period and were randomly allocated to one of the four feeding levels in the first period (Table 1).

**Table 1.** Animal groups (A, B, C and D) allocation to feeding levels (1·0, 0·8, 0·6 or 0·4 maintenance energy requirement (MER)) over the four experimental periods*

	Feeding levels (fractions of calculated MER)			
	1·0	0·8	0·6	0·4
Period 1	B	A	D	C
Period 2	C	B	A	D
Period 3	D	C	B	A
Period 4	A	D	C	B

* Groups each with three Boran steers.

### Diets and feeding

Experimental diets were based on an allocation of chaffed Rhodes grass late-cut hay (*Chloris gayana* cv. Boma DM: 875 g/kg; digestible energy: 8·4 MJ/kg DM; crude protein (CP): 73·1 g/kg DM), plus the addition of a small amount (equal to 10 % each of the calculated energy content of the ration) of cotton seed meal (DM: 947 g/kg; digestible energy: 12·7 MJ/kg DM; CP: 324·4 g/kg DM) and molasses (DM: 728 g/kg; digestible energy: 14·2 MJ/kg DM; CP: 46 g/kg DM) to the ration of animals being fed at 1·0 MER (in order to achieve required intake). Initially, MER was estimated using a regression equation developed from maintenance requirements for non-lactating dairy cattle^(21)^, and rations were formulated based on the proximate analysis of feed samples. The rations were subsequently reduced by 20 % after observing the animal response during pre-trial feeding; thus, the rations were set based on the following equation:
(1)
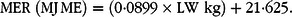


Treatment periods were 35 d, including an adaption period of 14 d prior to each measurement period. During the final 14 d of each period, total urine and faecal collection were undertaken and three, 22·5 h measurements of enteric CH_4_ production for each animal in every period were conducted on alternate days in open-circuit respiratory chambers. Because most animals were expected to lose weight during the experimental periods, each measurement period was followed by a 2-week ‘feed-up’ period where animals received 1 kg each of cotton seed meal and molasses in days 1–3, then 1·5 kg of each for days 4–14, plus *ad libitum* hay. Rations for animals in each experimental period were set on their LW on the first day of each experimental period, based on the animals’ estimated MER (eq. 1) and proximate analysis of the feeds as outlined above.

Animals were fed twice daily to decrease loss of hay through spillage (09.30 hours, after removal and weighing of orts and 14.00 hours), with hay placed in the feeding trough, and the supplement (1·0 MER only) in a separate bucket.

### Sample collection and analysis

#### Feed and refusals

After weighing, refusals were bulked daily by treatment, homogenised and a subsample (approximately 200 g) taken and stored in zipped polythene bags at −20°C. Samples of the basal diet were taken every 2 weeks and stored as for the refusals. At the end of each treatment period, the basal diet and refusals were bulked by treatment, homogenised and subsamples retained for subsequent processing and analysis.

Total faecal excretion was determined by daily collection over 6 d. Total faeces were weighed, and a sub-sample of approximately 500 g was transferred into labelled foil trays and then dried in a drying oven at 50°C until a constant weight was obtained for at least two consecutive days. Samples were cooled in desiccators, final weight determined, then stored in zipped polythene bags at room temperature until further analysis.

Dried faeces, feed and refusals were ground in a hammer mill through a 1 mm sieve and analysed as follows: true DM was determined at 105°C for 24 h; ash was determined by combustion in a muffle furnace at 550°C according to the methods of the AOAC (AOAC, 1990 methods no. 924.05). Feed and refusals were analysed for neutral-detergent fibre and acid-detergent fibre by the methods of Van Soest^(22)^ using an Ankom200 fibre analyzer (ANKOM Technology), with alfa-amylase enzyme. Total N content in feed and faeces was determined by the micro Kjeldahl procedure of AOAC (AOAC, 1990, method no. 988.05) with Se catalyst tablets. Gross energy content of feed was determined by bomb caliometry (Parr 6300, Parr Instruments). The digestible energy content of original fed samples was estimated from *in vitro* organic matter digestibility from the equation from the National Research Council^(24)^.

### Marker application procedures, analysis and calculations

Passage of liquid and solid digesta through the gastrointestinal tract was determined using Co-EDTA^(25)^ and Yb-marked fibre particles^(26)^, respectively. At commencement of passage rate determination, individual animals were offered a quantity of Yb-marked fibre equivalent to 560 mg Yb/kg LW^(27)^ mixed with 20 g molasses before morning feeding. After consumption of the marked fibre, each animal was drenched with Co-EDTA (23·56 mg Co/kg-LW). Time zero (t0) was taken as the completion of the Co-EDTA drench. To determine Yb and Co concentrations in faeces, about 60 g fresh faeces were sampled at 0 h (t_0_, then two hourly from 4 to 16 h after administration, four hourly from 16 to 40 h, six hourly from 40 to 88 h, then eight hourly from 88 to 136 h and finally at 148 h). Dried samples underwent sealed chamber digestion^(28)^, and Yb and Co concentrations were determined by inductively coupled plasma optical emission spectroscopy (ICP-OES 5100 VDV, Agilent Technologies). The cumulative quantity of Yb and Co excreted during the total collection period (148 h) was determined as the concentration of the respective elements in individual faecal samples multiplied by total faecal mass at time t_i_ (sampling time). PROC NLIN (method = dud) in SAS 9.1 (SAS Institute Inc.) was applied using the Type-N one-compartment Gamma-2 model of Richter & Schlecht^(27)^ for parameters of liquid (Co) and solid (Yb) digesta passage as follows: first appearance of markers (TT; equivalent to post-ruminal laminar flow) and ruminal passage rate (λ ruminal retention time (2λ−1).

### Enteric methane measurement and respiratory chamber operation

**Fig. 1.**
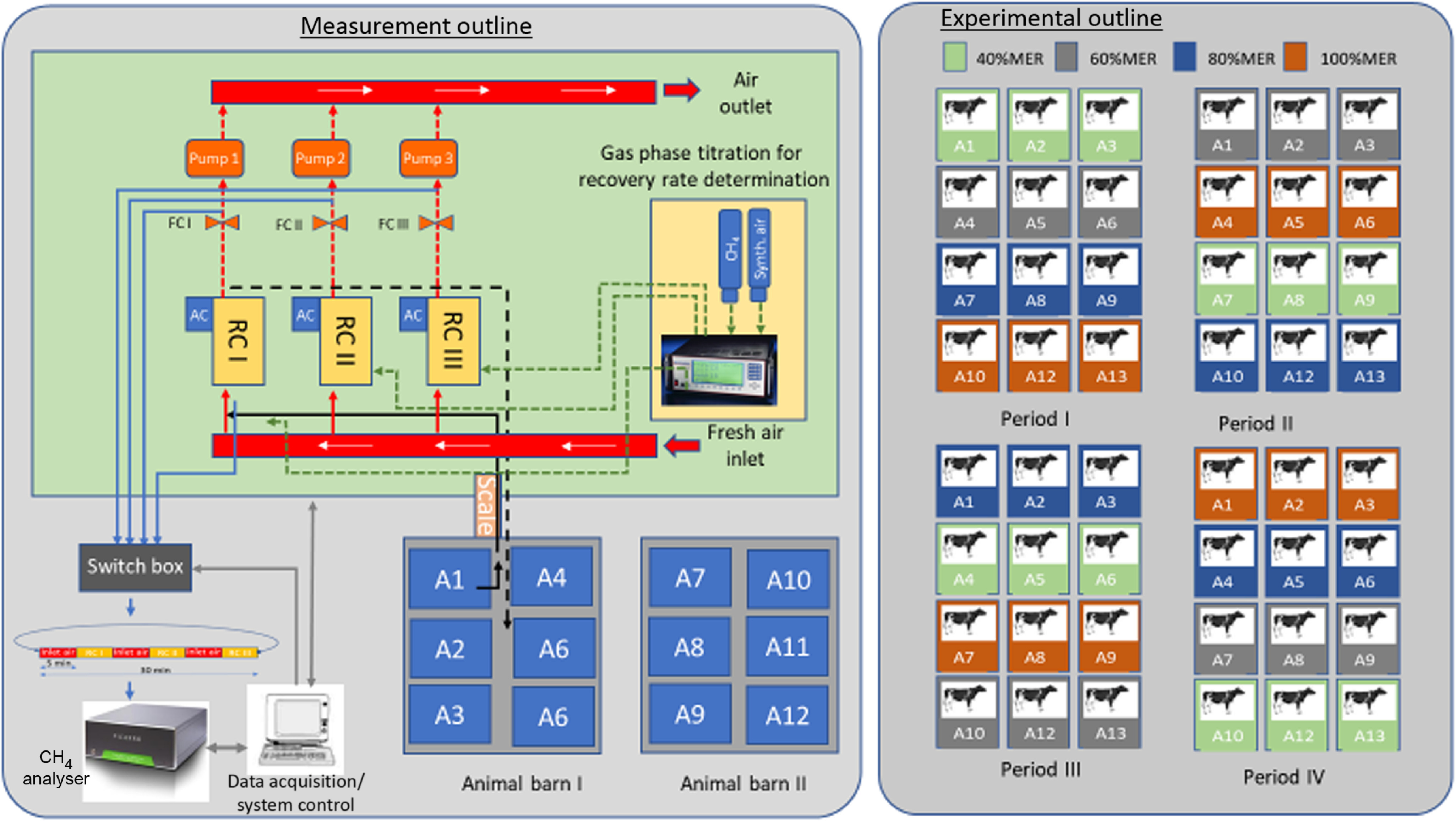
Schematic representation of the respiratory chambers arrangement, animal housing and treatment rotations over the four periods during the animal feeding trial. MER, maintenance energy requirement.

MPR was measured over three periods of approximately 22·5 h each, with each measurement period separated by 1 d; thus, six steers were assessed for MPR in each of weeks four and five in each experimental period (with total collection taking place in the alternate week) (see Fig. 1). Measurement was commenced at 09.30 hours with 1·5 h allocated for cleaning and unloading/loading animals. Thus, measurements were taken for all the steers over the same 22·5 h. On measurement days, immediately after refusals were collected, steers were taken from their pens to individual open-circuit respiratory chambers. The ration for the day was placed in the feed bin inside the chamber and the doors shut and sealed. Feed refusals were measured at the conclusion of each period, and steers were returned to their pens.

Respiration chambers, measuring equipment and operating conditions used in this study are presented here for the first time. Each custom-built chamber (3·08 m length × 1·50 m width × 2·00 m height; internal volume 8·90 m^3^) (No Pollution Industries) was constructed of injected polyurethane foam sandwich panels (60 mm thick), with external and internal lining of AISI 304 stainless steel. Two chambers shared a common wall, the third was independent. Large double-glazed, laminated glass panels were installed in side walls to provide visual communication between animals, with separate doors for entry and exit at opposite ends of each chamber also with windows fitted. Internally, chambers were equipped with supply and return ventilation grills, installed at ceiling height to mix the recirculation air and provide a slow movement of air across the chamber. The internal environment of each chamber was controlled by an air handling unit, treating a maximum volume of about 700 m^3^/h. Each air handling unit was equipped with a mixing, a filtering and a treatment section, composed of a cooling coil, a heating pack and a humidification circuit. A frequency-controlled main fan recirculates the air through the chamber and the air handling unit. Cold water from a chiller unit (MICS 0092 FF; Climaveneta Co.) passing through the cooling coil cooled and removed moisture from the air. The heating pack was electrically powered, and extraction of air from each chamber was achieved by a high-pressure blower (model: SCL K04-MS MOR, FPZ SpA), powered through a variable speed drive to allow control of the volume of air leaving each chamber, which was measured at the extraction point by a differential pressure system (model DP 2500-R8-AZ, Johnson controls Inc.), together with temperature and relative humidity. Overall control of the chambers, monitoring of conditions and events were carried out by custom designed software (Johnson controls metasys software, version 2.6, Johnson Controls Inc.), which integrates downloading capabilities for the main research parameters.

For the duration of the trial, the internal environment was set at 22°C, 50 % relative humidity and internal air circulation of 80 litres/s. Air was taken from the chambers under negative pressure by a pump at a flow of approximately 18 litres/s. MPR was calculated as chamber air flow multiplied by CH_4_ concentration in the chamber adjusted for CH_4_ concentration of the incoming air and temperature and atmospheric pressure in the chamber. The 22·5 h value was converted to MPR by multiplying by 24/22·5. Actual air flow through each chamber was measured using a venturi apparatus with differential pressure transducers (model DP 2500-R8-AZ, Johnson Controls Inc.).

Concentration of CH_4_ (parts per million per volume) was measured in the chamber incoming and exhaust air streams using a cavity ringdown laser absorption spectrometer (Picarro G2508 analyzer). The laser was validated using known CH_4_ concentrations. Sensors for relative humidity (model EE160 HCT01-00D, E+E Elektronic Ges.m.b.H, Elektronik Ges.m.b.H), CO_2_ (model EE85-10C35 E+E Elektronik Ges.m.b.H) and ambient temperature (model A99B-500C, Johnson Controls Inc.) monitored conditions in all chambers and recorded using the Sensing Science Laboratory software (Data Harvest Group) on a separate personal computer. Recoveries of CH_4_ were carried out for each chamber at the end of every experimental period by injecting known amounts of CH_4_ into the chamber using a gas-phase titration unit and measuring the total amount of CH_4_ found in the exhaust air.

### Statistical analysis

Firstly, the effects of treatment (intake level) on intake, digestibility, CH_4_ production and rumen kinetics were analysed using R 3.0.3 (R development core team). Treatment and period effects were compared using ANOVA type 3 using a linear mixed model fitted by restricted maximum likelihood (REML) *t* tests with Satterthwaite approximations of df (LmerTest) were used. Differences between means were compared using Tukey’s honestly significant difference, and level of significance was determined at 0·05. We also explored relationships in the data using a mixed linear model (lme4 in R^(29)^) using either a linear (Y = a + bX) or two-factor polynomial (Y = a(X)^2^ + bX + c) model for treatments with period and animal ID included as random variables; both models used maximum likelihoods with Satterthwaite approximations to df. The ‘best’ model was selected after examination of the *χ*^2^ values as well as the Akaike information criterion^(30)^, in order to assess if the extra complexity explained sufficient variability to justify its use. All linear and quadratic relationships are provided in online Supplementary Tables S1–S3.

#### Sample size justification

We hypothesised that digestibility would vary between the control (100 % MER) and the other treatment studied by 7–10 % of the overall mean. We then used a figure of 6·1 % in our calculations (from Doreau *et al.*^(31)^, with a standard deviation of 4·4 % of the mean. Using a standard two-tail *t* test comparison, 80 % statistical power, and level of significance set at 5 %, a total of nine animals per treatment was arrived at based on the formula described by Charan & Kantharia^(32)^. Because our experimental design was a 4 × 4 Latin square, we then settled on using three animals per treatment in each period which cumulatively gave us a total sample size of twelve at the end of the four periods. We used change in digestibility as our reference response variable instead of MPR because there were no experimental literatures exploring CH_4_ production at intakes below MER.

## Results

The basal Rhodes grass diet varied in organic matter between periods, albeit over a narrow range (911–929 (sem 0·32) g/kg DM; *P* < 0·05), but not in CP (*P* = 0·347). However, refusals, were lower in CP compared with rations fed (Table 2), indicating that animals were selecting for higher quality parts of the hay, but animals receiving the MER 0·4 and MER 0·6 treatments consistently consumed all of their allocated ration and had no refusals.

**Table 2.** Composition (DM, organic matter (OM) and crude protein (CP) of Rhodes grass hay and refusals fed to Boran steers at four fractions of calculated maintenance energy requirements (Mean values and pooled standard errors)

Components	Ration	Refusals	Pooled sem	*P*
DM (g/kg)	904·2	864·4	11·4	<0·05
OM (g/kg DM)	921·4	903·0	4·2	0·01
CP (g/kg DM)	67·4	46·3	0·7	<0·0001

Intakes differed between each treatment group as intended (Table 3), but intakes were lower in period 2 (*P* < 0·05) which was attributable to decreased intakes of the animals in 0·8 MER group, relative to the first period (*P* = 0·06). Animals in the 1·0 MER treatment group showed net weight gain, indicating that, even after adjusting the initial ration calculations, we overestimated MER, but the other three treatment groups lost weight in a linear manner (Table 3).

(2)
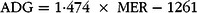


**Table 3.** Net intake as fed, and of DM, organic matter (OM) and crude protein (CP) plus average daily gain (ADG)* of Boran steers fed a ration consisting mainly of Rhodes grass hay offered at either 0·4, 0·6, 0·8 or 1·0 times calculated maintenance energy requirements (MER) over 21 d, following a 14 d adaptation period (Mean values and pooled standard errors)

Intake	1·0 MER	0·8 MER	0·6 MER	0·4 MER	Pooled sem	*P*
As fed (g)	5151^a^	4192^b^	3429^c^	2297^d^	68·9	<0·0001
DM (g)	4622^a^	3878^b^	3110^c^	2077^d^	51·4	<0·0001
OM (g)	4264^a^	3579^b^	2866^c^	1913^d^	48·9	<0·0001
CP (g)	407^a^	268^b^	210^c^	140^d^	3·4	<0·0001
ADG (kg)	0·200^a^	−0·100^b^	−0·310^c^	−0·715^d^	0·0611	<0·05

^a,b,c,d^ Mean values within a row with unlike superscript letters were significantly different (*P* < 0·05).

* Calculated over the full 35-d feeding period.

Intakes of the 1·0 and 0·8 MER groups were less during the 6 d collection and during the MPR measurement periods (*P* = 0·05) than intakes during the 21 d measurement period, but these differences were small (less than 2 %) and were not further considered (Table 4). However, there were no differences in faecal composition or apparent digestibility between any of the treatment groups, although there was a trend for MER 0·4 to have lower apparent digestibility of organic matter (Table 4).

**Table 4.** Intake, faeces and apparent DM digestibility (DMD), organic matter (OM) digestibility (OMD) and crude protein (CP) digestibility (CPD) of Boran steers fed a ration consisting mainly of Rhodes grass hay offered at either 0·4, 0·6, 0·8 or 1·0 times maintenance energy requirements (MER) measured over 6 d during a 21-d feeding period following a 14-d adaptation period (Mean values and pooled standard errors)

Intake	1·0 MER	0·8 MER	0·6 MER	0·4 MER	Pooled sem	*P*
As fed (g)	5043^a^	3969^b^	3539^c^	2385^d^	46·5	<0·001
DM (g)	4542^a^	3728^b^	3162^c^	2155^d^	64·3	<0·001
OM (g)	4194^a^	3442^b^	2968^c^	1987^d^	57·1	<0·001
CP (g)	403^a^	261^b^	218^c^	145^d^	3·6	<0·001
Faeces						
DM (g)	1·945^a^	1,546^b^	1,339^c^	929^d^	43·6	<0·05
OM (g)	1,674^a^	1,306^b^	1,174^c^	831^d^	41·9	<0·001
CP (g)	155·3^a^	99·6^b^	84·2^b^	61·0^c^	4·6	<0·001
Apparent digestibility						
DMD (g/100 g)	57·1	56·7	56·2	55·3	0·86	NS
OMD (g/100 g)	60·0	60·4	61·5*	56·6*	1·35	0·07
CPD (g/100 g)	61·5	60·4	60·0	56·5	1·54	NS

^a,b,c,d^ Mean values within a row with unlike superscript letters were significantly different (*P* < 0·05).

* Displayed a non-significant trend.

Recoveries of CH_4_ for individual chambers ranged from a mean of 96·3 to 102·7 % across the four measurement periods. Daily MPR differed between all treatment groups and showed a clear linear relationship to intake as anticipated (MPR = 109·8 × MER + 25·6; *P* < 0·001). Interestingly, MY increased modestly, also in a linear fashion as intakes and MPR fell further below 1·0 MER (MY = −5·23 × MER + 34·3; *P* = 0·001), with an increase of about 8 % (*P* < 0·001) in the 0·4 MER group compared with 1·0 MER (Table 5). CH_4_ produced per kg of digested organic matter (−10·25 × MER + 58·7; *P* < 0·001) and *Y*_*m*_ (*Y*_*m*_ = −1·81 × MER + 11·03) followed a similar trend, with increases of approximately 10 % in the 0·4 MER group compared with 1·0 MER (Table 5). In contrast, rumen MRT was prolonged in both liquid and solid phases, also in a linear fashion (MRT_liquid_ (h) = −11·6 × MER + 31·8; *P* < 0·001; MRT_solid_ (h) = −30 × MER + 92·0; *P* < 0·001) in all sub-maintenance groups by similar amounts of time, compared with the 1·0 MER treatment (Table 5).

**Table 5.** Methane production rate (MPR), methane yield (MY), methane produced/digested organic matter (MDOM), methane conversion factor (*Y_m_*) and rumen kinetics (mean retention time (MRT) (h) (liquid and solid phase)) of Boran steers fed a ration consisting mainly of Rhodes grass hay offered at either 0·4, 0·6, 0·8 or 1·0 times calculated maintenance energy requirements (MER) measured during a 21-d feeding period following a 14-d adaptation period (Mean values and pooled standard errors)

Enteric CH_4_	1·0 MER	0·8 MER	0·6 MER	0·4 MER	Pooled sem	*P*
MPR (g CH_4_/d)	133·3^a^	114·6^b^	94·4^c^	65·0^d^	3·55	<0·0001
MY (g CH_4_/kg DM intake)	29·0^a^	29·9^ab^	31·2^b^	31·2^b^	0·81	<0·001
MDOM (g CH_4_/kg DOM)	48·5^a^	50·6^ab^	51·8^bc^	53·4^c^	1·33	<0·001
*Y*_*m*_ (CH_4_ (MJ)/GEI (MJ))	9·14^a^	9·72^b^	9·74^b^	10·06^c^	0·225	<0·001
Rumen kinetics						
MRT liquid (h)	19·10^a^	23·28^b^	25·60^b^	26·19^b^	1·292	<0·005
MRT solid (h)	59·36^a^	72·05^b^	73·56^b^	78·86^b^	3·882	<0·001

GEI, gross energy intake.

^a,b,c,d^ Mean values within a row with unlike superscript letters were significantly different (*P* < 0·05).

## Discussion

The overarching aim of this study was to determine if cattle consuming rations at levels well below MER for maintenance produce more CH_4_ than would be expected from the amount of feed ingested. We initially estimated MER from the National Research Council^(21)^ recommendation for growing *B. taurus* cattle, subsequently decreasing allocations by 20 %, based on our empirical observations. However, the (nominally) 1·0 MER group still gained weight during the trial, and from (linear regression) analysis of LW records (eq. 2), we determined that our revised ration still overestimated MER by about 10 %. This is consistent with findings of a recent meta-analysis^(33)^ which suggests that the MER of *B. indicus × B. taurus* was up to 26 % less than that of pure *B. taurus* cattle. Ultimately, the miscalculation does not detract from the validity of the study, as it is clear from LW change that animals in all other treatment groups were in energy deficit and thus feed intake was below MER. Our thesis that both MY and *Y*_*m*_ would increase when cattle consume feed well-below MER was based partly on the idea that MRT is inversely related to feed intake, although differences in feed intake featured in the literature are most often related to the neutral-detergent fibre content of forage consumed *ad libitum*^(34)^. Prolonged residence time was observed in this study in both liquid and solid digesta fractions, but this was due to the restriction in intake rather than the limitation imposed by physical fill. It is perhaps important that the largest increase in MRT occurred between the 1·0 and 0·8 MER groups, with little prolongation seen past this. The larger difference in MRT between 1·0 MER and the other groups might be associated with 1·0 MER receiving cotton seed meal and molasses as part of their ration, but this is unproven, and even if it is the case, does not detract from the major findings of this study because the larger and significant changes to MY and *Y*_*m*_ occurred at the 0·4 and 0·6 levels. Thus, both MY and *Y*_*m*_ continued to increase as intake levels fell, but the changes were not accompanied by similar changes in MRT, nor by increases in apparent digestibility of the feed eaten. The observed results align with a study of Doreau *et al.*^(31)^, which found digestibility actually decreased at extremely low levels of intake and tellingly was not improved by additional rumen-degradable protein, indicating that reduced digestibility was probably not attributable to reduced rumen microbial activity. Instead, the amount of CH_4_ produced per unit of digested feed increased as feeding levels fell, indicating that a greater proportion of feed actually assimilated was being ultimately diverted to the production of CH_4_ – as suggested by the changes to both MY and *Y*_*m*_. The shift towards increased CH_4_ is consistent with a shift in volatile fatty acid production (towards acetate) and/or reduced production or refermentation of microbial protein, but this study does not furnish direct evidence of this.

Our findings differ from *Y*_*m*_ values currently in use, but other evidence exists to support their likely validity. We found that the *Y*_*m*_ of cattle fed at approximately maintenance (9·1 %) is higher than both Intergovernmental Panel on Climate Change recommendations (6·5 %) and recently published estimates^(8)^ of 6·3 % and our estimates of *Y*_*m*_ for cattle fed at 40 % of MER was greater still, at 10·1 %. However, another trial feeding tropical forages under *ad libitum* conditions reported a greater range of *Y*_*m*_ of 5·0–7·2 %^(35)^, with the highest values occurring when cattle were fed very low-quality grass, at what would have been effectively sub-maintenance requirements. Kaewpila & Sommart^(36)^ calculated (from earlier studies) a *Y*_*m*_ of 8·2 % for *B. indicus* cattle fed low-quality forage – but a limitation of this study is that it does not seek to separate the possible influences of feeding levels from feed quality. While each of the studies detailed above, as well as the one reported here, has been conducted using cattle of predominantly *B. indicus* origins, it could be tempting to attribute the higher *Y*_*m*_ values to breed effects. However, we assert that we have clearly demonstrated the contribution of feeding levels apart from any genetic effects that might exist. To clarify this, it would be desirable to conduct further measurements of MPR in cattle consuming tropical forages *ad libitum* to determine the effect on *Y*_*m*_ values.

### Conclusion

Feeding low-quality tropical forages at restricted intakes substantially increase both the MY and *Y*_*m*_ of the consumed forages, whilst severe intake restriction exacerbates the case by a further 8–10 %. While meriting further investigation to confirm, we believe these changes observed in animals fed well below their voluntary intake are quantitatively important for both greenhouse gas inventory and animal husbandry in tropical rain-fed systems. Firstly for ruminants in areas where there are marked seasonal nutritional deficits such as in Sub-Saharan Africa (and northern Australia), incorporation of a higher *Y*_*m*_ value (as suggested here) for part of the year would increase calculated emission factors by 10 % or more, which may significantly alter current estimates of CH_4_ emissions from livestock, for example, in Africa^(9)^. More importantly, perhaps, is that this work suggests that improving the feeding of ruminants under nutritional stress has not only the potential to reduce their contribution to greenhouse gas emissions but also direct economic benefits for farmers via improved nutrient partitioning away from the production of CH_4_ towards growth.

## Supplementary material

For supplementary material accompanying this paper visit http://dx.doi.org/10.1017/S0007114519003350. click here to view supplementary material
