# Supplementary material for: Severe below-maintenance feed intake increases methane yield from enteric fermentation in cattle
Source: Br J Nutr. 2020 Mar 25;123(11):1239–46. doi: 10.1017/S0007114519003350 (PMC7512143; doi:10.1017/S0007114519003350)
Supplement: Supplementary file 1 [file S0007114519003350sup001.docx]

**Supplementary Table 1. Linear mixed model coefficients, log likelihoods, Akaike’s Information Criterion (AIC) and Chi square for model comparison for net intake as fed, and of dry matter (DM), organic matter (OM) and crude protein (CP) plus average daily gain (ADG)^#^ of Boran steers fed a ration consisting mainly of Rhodes grass hay offered at either 0.4, 0.6, 0.8 or 1.0 times calculated Maintenance Energy Requirements (MER) over 21d, following a 14 d adaptation period.**

| Intake | Linear model coefficients: y = a + bx | | Log  likelihood | AIC | Polynomial model coefficients:  y = a + bx + cx^2^ | | | Log  likelihood | AIC | Model  Comparison  (Chi square) |
| --- | --- | --- | --- | --- | --- | --- | --- | --- | --- | --- |
|  | a | b |  |  | a | b | c |  |  |  |
| As Fed (g) | **503^***^** | **4662^***^** | -332.7 | 675.4 | 28.2 | 6175.6^***^ | -1081.1 | -331.6 | 675.3 | 0.1442 |
| DM (g) | 480^***^ | 4201^***^ | -317.4 | 644.8 | **-316.3** | **6736^***^** | **-1811^***^** | -310.5 | 633.0 | < 0.001 |
| OM (g) | 438^***^ | 3883^***^ | -313.8 | 637.6 | **-295.6** | **6216^***^** | **-1666^***^** | -306.7 | 625.5 | < 0.001 |
| CP (g) | -43.9^***^ | 428.6^***^ | -219.4 | 448.9 | **144.4^***^** | **-170.6^*^** | **428.0^***^** | -200.2 | 412.3 | < 0.001 |
| ADWG (kg) | **-1.261^***^** | **1.474^***^** | 12.9 | -15.8 | -1.576^***^ | 2.478^**^ | -0.718 | 13.7 | -15.4 | 0.2073 |

Note: Significance codes: “***” P<0.001; “**” P<0.01; “*” P<0.05; “.” P<0.10. Bolded coefficients indicate the preferred model (either linear or 2-factor polynomial)

**Supplementary Table 2. Linear mixed model coefficients, log likelihoods, Akaike’s Information Criterion (AIC) and Chi square for model comparison for Intake, faeces and apparent digestibility of dry matter (DMD), organic matter (OMD) and crude protein (CPD) of Boran steers fed a ration consisting mainly of Rhodes grass hay offered at either 0.4, 0.6, 0.8 or 1.0 times Maintenance Energy Requirements (MER) measured over 6d during a 21d feeding period following a 14 d adaptation period .**

| Intake | Linear model coefficients: y = a + bx | | Log  likelihood | AIC | Polynomial model coefficients:  y = a + bx + cx^2^ | | | Log  likelihood | AIC | Model  Comparison  (Chi square) |
| --- | --- | --- | --- | --- | --- | --- | --- | --- | --- | --- |
|  | a | b |  |  | a | b | c |  |  |  |
| As Fed (g) | **791^***^** | **4210^***^** | -347.2 | 704.4 | 520 | 5072^*^ | -615 | -347.1 | 706.2 | 0.6561 |
| DM (g) | **692^***^** | **3865^***^** | -337.7 | 685.4 | 135 | 5640^***^ | -1266 | -337.0 | 686.0 | 0.2499 |
| OM (g) | **664^***^** | **3550^***^** | -333.6 | 677.3 | 1.9 | 5657^***^ | -1504 | -332.5 | 677.1 | 0.1376 |
| CP (g) | -29.3^*^ | 409.7^***^ | -225.2 | 460.3 | **157.4^***^** | **-184.5** | **424.0^***^** | -215.5 | 442.9 | < 0.001 |
| Faeces | | | | | | | | | | |
| DM (g) | **300.0^**^** | **1628^***^** | -319.4 | 648.7 | 269.5 | 1725.1 . | -69.2 | -319.4 | 650.7 | 0.916 |
| OM (g) | **273.0^**^** | **1365^***^** | -315.9 | 641.8 | 537.9 . | 522.2 | 602.0 | -315.4 | 642.8 | 0.310 |
| CP (g) | -4.38 | 149.1^***^ | -210.3 | 430.6 | **84.78^**^** | **-134.6** | **202.6^**^** | -204.9 | 421.9 | 0.001 |
| Apparent digestibility | | | | | | | | | | |
| DMD (g/100g) | 54.25^***^ | 2.92 . | -113.7 | 237.3 | 53.04^***^ | 6.76 | -2.74 | -113.6 | 239.2 | 0.752 |
| OMD (g/100g) | 56.40 | 4.60 | -140.8 | 291.6 | **41.24^***^** | **52.84^*^** | **-34.42^*^** | -138.8 | 289.6 | 0.046 |
| CPD (g/100g) | **54.18^***^** | **7.69^*^** | -145.0 | 300.1 | 47.54^***^ | 28.83 | -15.08 | -144.7 | 301.5 | 0.431 |

Note: Significance codes: “***” P<0.001; “**” P<0.01; “*” P<0.05; “.” P<0.10. Bolded coefficients indicate the preferred model (either linear or 2-factor polynomial)

**Supplementary Table 3. Linear mixed model coefficients, log likelihoods, Akaike’s Information Criterion (AIC) and Chi square for model comparison for methane production rate (MPR) methane yield (MY) methane produced/digested organic matter (MDOM), methane conversion factor (Ym) and rumen kinetics (mean retention time MRT (h) [liquid and solid phase) of Boran steers fed a ration consisting mainly Rhodes grass hay offered at either 0.4, 0.6, 0.8 or 1.0 times calculated Maintenance Energy Requirements (MER) measured during a 21d feeding period following a 14 d adaptation period**

| Intake | Linear model coefficients: y = a + bx | | Log  likelihood | AIC | Polynomial model coefficients:  y = a + bx + cx^2^ | | | Log  likelihood | AIC | Model  Comparison  (Chi square) |
| --- | --- | --- | --- | --- | --- | --- | --- | --- | --- | --- |
|  | a | b |  |  | a | b | c |  |  |  |
| MPR | **19.4***** | **82.3***** | -172.6 | 355.2 | 1.30 | 140.0*** | -41.2 | -171.3 | 354.5 | 0.104 |
| MY | **25.8***** | **-4.03***** | -102.2 | 214.5 | 25.7*** | -3.42 | -0.44 | -102.2 | 216.5 | 0.936 |
| MDOM | **44.35***** | **-8.50***** | -137.0 | 284.0 | 49.7*** | -25.6 | 12.2 | -136.5 | 285.0 | 0.321 |
| Ym | **8.31***** | **-1.40***** | -37.4 | 84.9 | 8.33*** | -1.44 | -0.03 | -37.4 | 86.9 | 0.981 |
| Rumen kinetics | | | | | | | | | | |
| MRT liquid | **31.8^***^** | **-11.8^***^** | -132.8 | 275.6 | 21.9^***^ | 19.6 | -22.4^*^ | -130.4 | 272.8 | 0.029 |
| MRT solid | **92.0^***^** | **-30.0^***^** | -182.4 | 374.7 | 71.6^***^ | 34.7 | -46.2 | -181.1 | 374.3 | 0.118 |

Note: Significance codes: “***” P<0.001; “**” P<0.01; “*” P<0.05; “.” P<0.10. Bolded coefficients indicate the preferred model (either linear or 2-factor polynomial)
